# Supplementary material for: Predicting visual function by interpreting a neuronal wiring diagram
Source: Nature. 2024 Oct 2;634(8032):113–23. doi: 10.1038/s41586-024-07953-5 (PMC11446822; doi:10.1038/s41586-024-07953-5)
Supplement: Supplementary file 6 — CRF and ERF predictions for individual TmY4 and TmY9 cells. Analogous to Supplementary Data 3, but for TmY target types. Shown are the top four monosynaptic pathways, the strongest pathway passing through each of the top ten intermediary types (ranking from Extended Data Fig. 7), and the trisynaptic pathway Tm1–TmY–Dm3–TmY (see the section entitled Prediction of spatial normalization). [file 41586_2024_7953_MOESM6_ESM.zip › DataS4/TmY9qΓèÑ/720575940628774662.pdf]

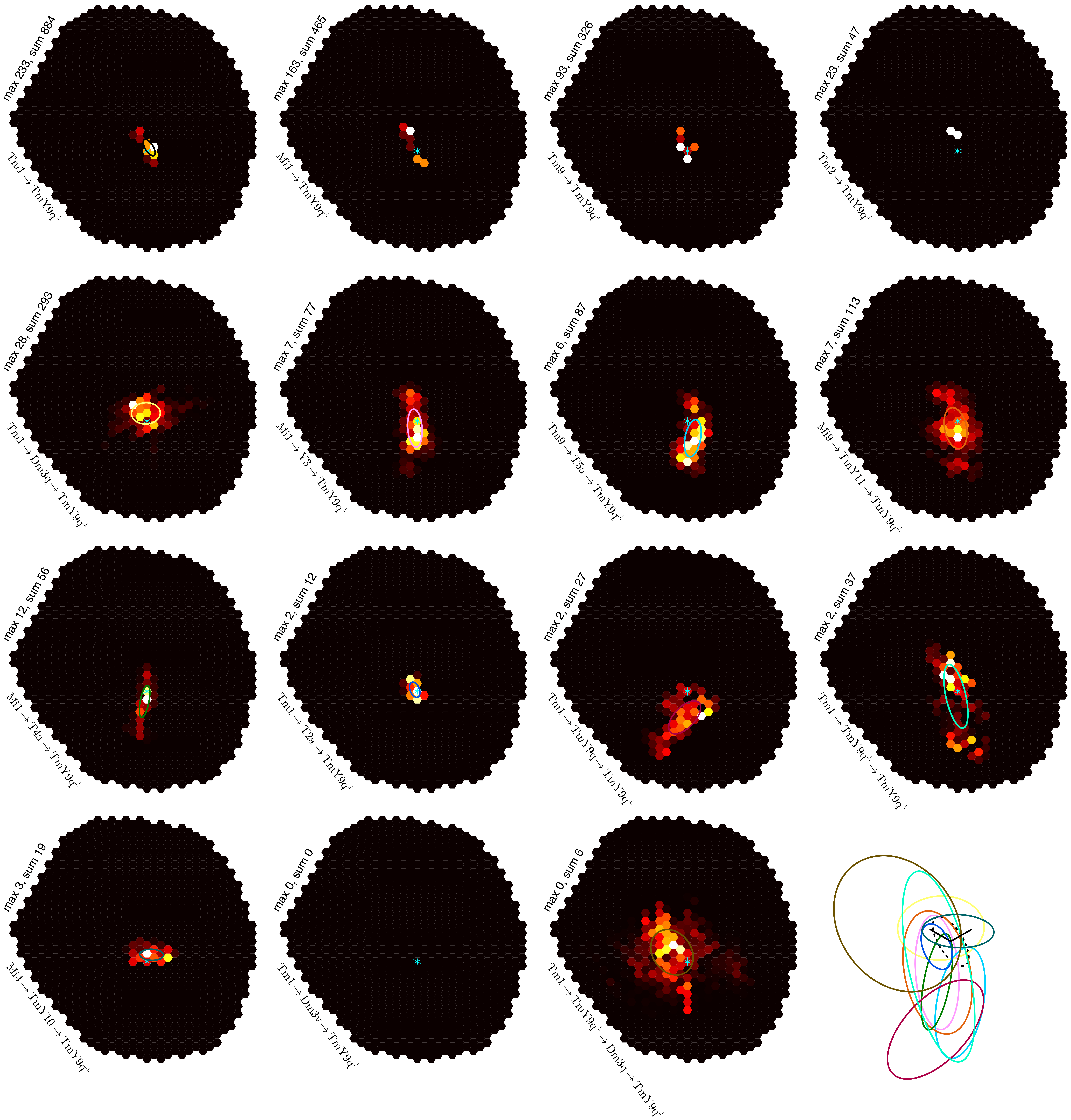

max 233, sum 884
Tm1 → TmY9q⊥
max 163, sum 465
Mi1 → TmY9q⊥
max 93, sum 326
Tm9 → TmY9q⊥
max 23, sum 47
Tm2 → TmY9q⊥
max 28, sum 293
Tm1 → Dm3q → TmY9q⊥
max 7, sum 77
Mi1 → Y3 → TmY9q⊥
max 6, sum 87
Tm9 → T5a → TmY9q⊥
max 7, sum 113
Mi9 → TmY11 → TmY9q⊥
max 12, sum 56
Mi1 → T4a → TmY9q⊥
max 2, sum 12
Tm1 → T2a → TmY9q⊥
max 2, sum 27
Tm1 → TmY9q → TmY9q⊥
max 2, sum 37
Tm1 → TmY9q⊥ → TmY9q⊥
max 3, sum 19
Mi4 → TmY10 → TmY9q⊥
max 0, sum 0
Tm1 → Dm3v → TmY9q⊥
max 0, sum 6
Tm1 → TmY9q⊥ → Dm3q → TmY9q⊥
